# Alcohol intake and cause-specific mortality: conventional and genetic evidence in a prospective cohort study of 512,000 adults in China

**DOI:** 10.1016/S2468-2667(23)00217-7

**Published:** 2023-11-21

**Authors:** Iona Y Millwood, Pek Kei Im, Derrick Bennett, Parisa Hariri, Ling Yang, Huaidong Du, Christiana Kartsonaki, Kuang Lin, Canqing Yu, Yiping Chen, Dianjianyi Sun, Ningmei Zhang, Daniel Avery, Dan Schmidt, Pei Pei, Junshi Chen, Robert Clarke, Jun Lv, Richard Peto, Robin G Walters, Liming Li, Zhengming Chen

**Affiliations:** 1 Clinical Trial Service Unit and Epidemiological Studies Unit (CTSU), Nuffield Department of Population Health, University of Oxford, Oxford, UK; 2 Medical Research Council Population Health Research Unit (MRC PHRU), Nuffield Department of Population Health, University of Oxford, Oxford, UK; 3 Turku PET Centre, Turku University Hospital and University of Turku, Turku, Finland; 4 Department of Epidemiology and Biostatistics, School of Public Health, Peking University, Beijing, China; 5 Peking University Center for Public Health and Epidemic Preparedness & Response, Beijing, China; 6 Key Laboratory of Epidemiology of Major Diseases (Peking University), Ministry of Education, Beijing, China; 7 NCDs Prevention and Control Department, Sichuan CDC, Chengdu, Sichuan, China; 8 China National Center for Food Safety Risk Assessment, Beijing, China

## Abstract

**Background:**

Genetic variants strongly influencing alcohol use in East Asians can help assess the causal effects of alcohol consumption on cause-specific mortality.

**Methods:**

The prospective China Kadoorie Biobank enrolled 512,724 adults aged 30-79 years from ten areas during 2004-2008, and recorded 56,550 deaths during 12-years follow-up, including 23,457 deaths among 168,050 participants genotyped for *ALDH2*-rs671 and *ADH1B*-rs1229984. Adjusted hazard ratios (HR) for cause-specific mortality by self-reported and genotype-predicted alcohol intake were estimated using Cox regression.

**Findings:**

Among men, 33% drank alcohol in most weeks. In conventional observational analyses, compared with moderate drinkers, ex-, non-, and heavier drinkers had higher risks of death from most major causes. Among current drinkers, higher alcohol intake was associated with higher mortality risks from cancers, CVD, liver diseases, non-medical causes, and all-causes (HRs 1.18, 1.19, 1.51, 1.15 and 1.18 per 100 g/week, respectively). In men, *ALDH2*-rs671 and *ADH1B*-rs1229984 genotypes predicted 60-fold differences in mean alcohol intake. Genotype-predicted alcohol intake was linearly and positively associated with risks of death from all-causes (n=12939; HR=1.07, 95%CI=1.05−1.10) and from pre-defined alcohol-related cancers (n=1274; 1.12, 1.04−1.21), liver diseases (n=110; 1.31, 1.02−1.69), and CVD (n=6109; 1.15, 1.10−1.19), chiefly due to stroke (n=3285; 1.18, 1.12-1.25) rather than IHD (n=2363; 1.07, 1.00-1.15). Results were largely consistent using a polygenic score to predict alcohol intake, with no departure from linearity for alcohol-related cancers, CVD and all-cause mortality across low to high alcohol intake strata. Among women, ~2% drank alcohol, and although power was low to assess observational associations of alcohol with mortality, the genetic evidence suggested that the excess risks in men were due to alcohol not pleiotropy.

**Interpretation:**

Higher alcohol intake linearly increased the risks of death overall and from major diseases in Chinese men. There was no genetic evidence of protection with moderate drinking for all-cause and cause-specific mortality, including CVD.

**Funding:**

Kadoorie Charitable Foundation, National Natural Science Foundation of China, British Heart Foundation, Cancer Research UK, GlaxoSmithKline, Wellcome Trust, and Medical Research Council, Chinese Ministry of Science and Technology

## Background

Worldwide the harmful use of alcohol accounted for an estimated ~3 million deaths in 2016.^1^ The main alcohol-attributed causes of death include liver cirrhosis, cardiovascular disease (CVD), certain cancers (e.g. mouth and throat, oesophagus, liver), tuberculosis, pneumonia, mental health problems, and injuries.^1,2^ Estimates of the disease burden attributed to alcohol intake have been typically based on risk estimates derived from observational studies of predominantly Western populations. A recent study highlighted the importance of evidence from diverse populations, with different region- and age-specific disease rates.^3^ Furthermore, although the observational evidence is based on large sample sizes, the associations observed may not reflect causal effects, which could affect estimation of the adverse effects of alcohol use.^4^

Over the last few decades, meta-analyses of prospective studies report that the observed risks of mortality overall, and particularly from CVD, were lower among moderate drinkers, leading to widespread acceptance of ~1-2 drinks/day as a safe level of consumption in general populations.^5–7^ However, systematic differences in health characteristics and behaviours (such as prior ill health, socio-economic status, or smoking behaviours) between non-drinkers, moderate and heavy drinkers, often influenced by selection into cohort studies and their demographics characteristics, can lead to reverse causation (where health status affects drinking patterns), confounding and other biases.^7,8^ In China, where alcohol consumption has increased steadily in recent decades, there is limited evidence available on alcohol drinking and cause-specific mortality in the general adult population.^9,10^

Mendelian randomisation (MR) uses genetic variants as instrumental variables to assess the causal relevance of alcohol intake while minimising the biases inherent in conventional observational studies.^11^ An MR study of European ancestry individuals associated alcohol intake with a higher risk of all-cause mortality, but specific causes of death were not investigated, nor was the shape of the association across different levels of intake.^12^ In East Asian populations, two common genetic variants (*ALDH2*-rs671 and *ADH1B*-rs1229984) alter the function of enzymes involved in alcohol metabolism and strongly affect alcohol tolerability and alcohol intake.^4^ These genetic variants have been used to assess the causal relevance of alcohol intake for incidence of CVD and other diseases, and overall mortality.^4,13,14^ Ascertaining the causal relevance of alcohol for major causes of death, particularly CVD where associations of alcohol with fatal compared with non-fatal events may differ, can improve estimations of the global burden of alcohol use and inform policies for prevention of alcohol-related harms.

This study investigated the associations between alcohol consumption and cause-specific mortality among >512,000 adult men and women from the prospective China Kadoorie Biobank (CKB). In addition to assessing conventional observational associations, we used an MR approach to assess the strength, shape and causal relevance of genotype-predicted alcohol intake with cause-specific mortality among a subset of >168,000 men and women with data on *ALDH2*-rs671 and *ADH1B*-rs1229984 genotype. Additional analyses used a polygenic score to predict alcohol intake and evaluate linearity of the associations with mortality.

## Methods

### Study design and participants

CKB is a prospective cohort of 512,724 adults aged 30-79 years and without major disability at enrolment (response rate 28%) during 2004-2008 from ten areas of China.^15^ At baseline, participants attended survey clinics and completed an interviewer-administered laptop-based questionnaire covering socio-demographic and lifestyle characteristics (e.g. smoking, alcohol drinking) and medical history. Physical measurements were taken (e.g. blood pressure, anthropometry), and a 10 ml blood sample was collected. Resurveys of ~5% of surviving participants, following similar procedures, were undertaken in 2008 (n=19,786), 2013-14 (n=25,041), and 2021-22 (n=25,087). Ethics approval was obtained from local, national and international ethics committees and all participants provided written informed consent.

### Assessment of alcohol drinking

Alcohol drinking patterns were self-reported at baseline and resurveys.^16,17^ Participants were classified as current drinkers (some alcohol use in most weeks in the past year), non-drinkers (no alcohol use in the past year and never drank in most weeks), occasional drinkers (occasional alcohol use in the past year and never drank in most weeks), and ex-drinkers (occasional or no alcohol use in the past year but previously drank in most weeks). Current drinkers provided further details about their drinking patterns (including frequency, amount, beverage type), and were further classified by weekly intake (<140, 140-279, 280-419, 420+ g/week for men; <70, 70+ g/week for women). To account for measurement error and within-person variability in self-reported alcohol use over time, for each of these baseline-defined groups the usual mean level of alcohol intake of the group was estimated from the average of intakes at two resurveys (**appendix p8**).^18^

### Follow-up for cause-specific mortality

Cause-specific mortality was ascertained through linkage via unique national identification number to local death registries managed by China Centre for Disease Control (CDC). All deaths were reviewed by regional CDC staff and the underlying cause of death was assigned using the International Classification of Diseases, tenth revision (ICD-10). By 1.1.2019, after median 12 years follow-up (interquartile range 11-13), 56,550 (11%) participants had died, and 4,028 (1%) were lost to follow-up.

For the present study, deaths were grouped into broad categories (e.g. CVD ICD-10 chapter I00-I99), specific causes (e.g. IHD ICD-10 I20-I25), or by previously assigned relationship to alcohol (e.g. cancers or other diseases and injuries designated as related to alcohol by IARC or WHO) (**appendix p9**).^2,19^

### Genotyping and estimation of genotype-predicted mean alcohol intake

168,050 participants were genotyped for *ALDH2*-rs671 and *ADH1B*-rs1229984, including 151,347 randomly-selected (included in all genetic analyses), and 16,703 who had been selected for nested case-control studies of CVD or COPD (only included as cases in analyses of relevant outcomes) (**appendix p10**).

Using a previously-described approach, alcohol intake was predicted using a combination of genotype and study area, both of which had strong associations with alcohol intake, enabling a wide range of alcohol intake levels to be assesed.^4^ Mean alcohol intake was calculated among men within each of the 90 combinations of genotypes (*ALDH2*-rs671 and *ADH1B*-rs1229984 each AA, AG or GG, resulting in nine combined genotypes) across the ten areas. Thresholds at 10, 25, 50, 100, and 150 g/week, were applied to group the genotype-predicted mean alcohol intake into six categories (C1-C6) for genetic analyses among all genotyped participants. Combining genotype with study area enabled a reliable assessment of the shape and strength of associations with outcomes across a wide range of genotype-predicted mean alcohol intake, rather than the smaller range predicted by the genotypes alone.

Women were assigned into the same six categories as men based on their genotype and area, without reference to their mean alcohol intake, to assess potential pleiotropic effects of the genotypes studied ie, effects of genotype not mediated by alcohol.

Supplemental analyses among 85,386 men and women used a weighted polygenic score of 825 alcohol-related variants from a multi-ancestry genome-wide meta-analysis to predict alcohol intake.^20^

See **Supplementary Methods** for details.

### Statistical methods

Analyses were conducted among men and women separately. In conventional observational analyses, Cox proportional hazards regression models were stratified for age-at-risk (5-year groups from 35-84 years) and ten areas, and adjusted for education, household income, smoking, physical activity, and fresh fruit intake. Participants reporting prior diseases at baseline were excluded. To allow comparisons in analyses involving more than two exposure groups, the variance of the log risk in each group, including the reference group, was calculated to obtain group-specific 95% CIs.^21^ To account for measurement error and within person variability in alcohol use over time (i.e. regression dilution bias), among current drinkers the log HRs were plotted against usual alcohol intake.^18^ The slope of a weighted linear regression through the plotted log HRs was used to estimate the HR per 100 g/week (~1-2 drinks/day, assuming 1 drink=10g alcohol) usual alcohol intake. Sensitivity analyses excluded the first five years of follow-up and additionally adjusted for red meat intake and self-rated health.

In genetic analyses, associations of genotype-predicted alcohol categories with alcohol intake and with potential confounders were assessed. Cox proportional hazards regression models were stratified for age-at-risk and ten areas, and adjusted for genomic principal components (PCs).^22^ Log HRs were plotted against mean alcohol intake in each genotype-predicted alcohol intake category. To estimate the HR per 280 g/week, analyses were performed separately within each area with adjustment for age-at-risk and regional PCs. The slopes of a weighted linear regression within each area were meta-analysed with inverse-variance weighting (IVW-MA). To assess potential pleiotropy of the genetic instrument, a heterogeneity test compared the meta-analysed slopes between men and women. Sensitivity analyses included adjusting for covariates; excluding prior diseases; using logistic regression or a two-stage least-square (2SLS) MR approach; using the 90 genotype-area combinations as a continuous exposure; and excluding the highest category of predicted alcohol intake.^23^ Analyses of the individual genetic variants included a comparison of GG vs. GA genotypes, and interaction between genotypes and self-reported alcohol intake.

Supplemental analyses with a polygenic score used a 2SLS approach within areas, followed by IVW-MA. Beta estimates from the regression of alcohol against the polygenic score in men were applied to the polygenic score values in women, to facilitate an assessment of pleiotropy. Non-linear MR stratified participants by average alcohol intake levels using the doubly-ranked method.^24^ Local Average Causal Effects (LACE) within strata calculated the ratio of the associations of the polygenic score with alcohol intake (log-transformed) and with mortality. Fractional polynomial smoothing was used to generate risk curves. Sensitivity analyses included use of the residual method for stratification.^24^

Since all-cause mortality is a competing risk for cause-specific mortality, Cox regression models censored participants at death from any cause (or loss to follow-up or the global censoring date 1.1.2019) to estimate cause-specific HRs, which compared event rates in participants who were alive and free of the event of interest. Comparing the HRs for the first 6 and subsequent years of follow-up, showed no evidence of departure from the proportional hazards assumption, apart from liver disease deaths in genetic analyses, with greater HRs in the earlier follow-up period (p-heterogeneity=0.002).

See **Supplementary Methods** for details of statistical methods.

Analyses used R software (version 4.0.5).

### Role of the funding source

The funders had no role in the study design, data collection, data analysis and interpretation, writing of the manuscript, or the decision to submit the article for publication.

## Results

Among 512,724 study participants, the mean age at baseline was 52 years (SD 11), 210,205 (41%) were men and 226,191 (44%) were from urban areas. Among men, 69,900 (33%) reported drinking alcohol in most weeks (current drinkers), which varied across the ten study areas (Table 1; **appendix p11**). Non- and ex-drinkers were older than occasional and current drinkers, were more likely to live in rural areas, and had poorer health at baseline. Education and household income levels were highest among moderate drinkers (up to 140 g/week). Heavier drinkers were more likely to smoke, and consumed fresh fruit less frequently. Alcohol was consumed mainly as spirits, and with meals, and 18% of current drinkers reported flushing after drinking (**appendix p12**). Among 302,519 women, 101,285 (33%) drank alcohol occasionally, but only 6244 (2%) were current drinkers.

### Genetic associations with alcohol intake and confounders

The A-alleles of *ALDH2*-rs671 (frequency 0.21, range by area 0.13-0.29) and *ADH1B*-rs1229984 (0.69, 0.64-0.74) (**appendix p13**), were both associated with lower alcohol intake (**appendix p14**). Mean alcohol intakes among men with AA, AG and GG genotypes, respectively, were 2, 37 and 162 g/week for *ALDH2*-rs671, and 101, 109 and 162 g/week for *ADH1B*-rs1229984. Combining the two variants with area predicted 60-fold differences in mean alcohol intake in men, from 4 g/week in the lowest to 255 g/week in the highest category, with the prevalence of ever-regular drinking ranging from 3% (124/4269) to 74% (11,720/15,838) (**appendix p15-16**). These categories were not associated with education, smoking, or other potential confounders, except for fresh fruit intake, which was lower in the higher alcohol intake categories. Among women, similar genotype-area categories were not associated with appreciable differences in mean alcohol intake (range 1-8 g/week), or potential confounders.

### Associations of self-reported alcohol intake with mortality

Of the 56,550 deaths recorded (31,956 in men and 24,594 in women), CVD (23,290 deaths) and cancers (17,691) together accounted for 72%, with respiratory diseases (5,362), and non-medical causes (3,750) accounting for a further 16% of deaths (**appendix p17**).

**Figure 1:**
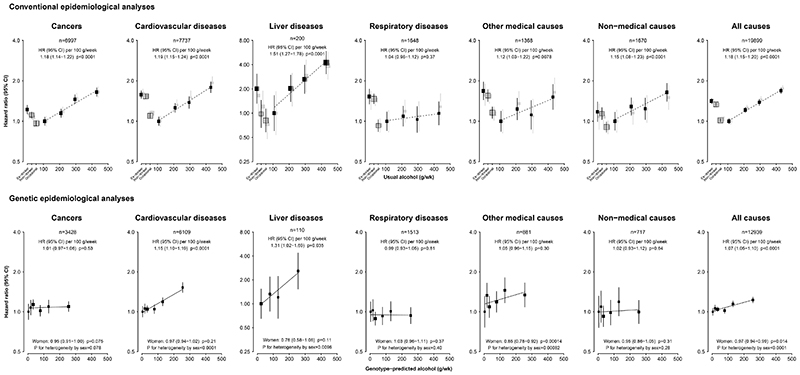
Conventional and genetic associations of alcohol intake with major cause-specific and all-cause mortality, in men Conventional epidemiological analyses relate self-reported drinking patterns at baseline to mortality from major causes (all major causes are shown except for infectious diseases where numbers of deaths were lower) and all-causes. Current drinkers with the lowest mean alcohol intake are the reference group. The black squares represent findings from the main model adjusted for age-at-risk, area, education, household income, smoking, physical activity, and fresh fruit intake, with exclusion of participants with prior chronic disease. The HRs for current drinkers are plotted against usual alcohol intake and a weighted linear regression through the plotted estimates gives the HR (95% CI) per 100 g/week (~1-2 drinks/day, assuming 1 drink contains 10g alcohol). The grey squares represent findings from sensitivity analysis which further exclude the first five years of follow-up. Genetic epidemiological analyses relate mean alcohol intake in six categories of genotype-predicted intake to mortality from major causes. The lowest mean intake group is the reference, and analyses are adjusted for age-at-risk, area and genomic national principal components. HRs are plotted against the mean alcohol intake in each category. The HR (95% CI) per 100 g/week is the inverse-variance-weighted mean of a weighted linear regression through the plotted estimates within each study area, adjusted for age-at-risk, and genomic regional principal components. The HR (95% CI) across six genetic categories in women applied the mean male intakes for each category, and the heterogeneity of effects was compared between men and women, to assess pleiotropy. The HR is plotted on a log scale. Each box represents HR with the area inversely proportional to the variance of the group-specific log hazard within each subplot. The vertical lines indicate group-specific 95% CIs. HR: hazard ratio; CI, confidence interval.

Among men, there were J-shaped or U-shaped associations between self-reported alcohol consumption and major causes of death, with higher risks in ex- and non-drinkers and heavier drinkers, compared with occasional or moderate drinkers, in analyses adjusted for age-at-risk, area, education, household income, smoking, physical activity, and fresh fruit intake (Figure 1; **appendix p18**). Consistent with the J-shaped association with all-cause mortality, the estimated survival rate was higher in occasional and current drinkers, compared with non- and ex-drinkers (**appendix p19**).

Among current drinkers, mortality risks increased with higher usual alcohol intake for CVD (HR per 100 g/week 1.19, 95% CI 1.15−1.24), cancers (1.18, 1.14−1.22), liver diseases (1.51, 1.27−1.78), other (including ill-defined) medical causes (1.12, 1.03−1.22), non-medical causes (1.15, 1.08−1.23), and all-causes (1.18, 1.15−1.20). The amount of alcohol intake was not associated with risks of death from infectious or respiratory diseases. With finer division of alcohol intake, the risk of all-cause mortality increased in a dose-response, with no evidence of a threshold at lower intakes (**appendix p20**).

**Figure 2:**
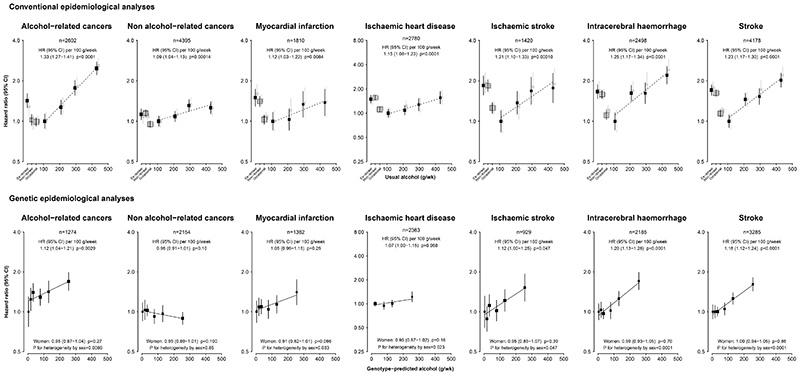
Conventional and genetic associations of alcohol intake with mortality from aggregated cancers and cardiovascular disease types, in men Alcohol-related cancers: Lip, oral cavity, pharynx, larynx, oesophagus, liver, colon-rectum, and female breast, defined as related to alcohol by the International Agency for Cancer Research (IARC).Conventions as Figure 2.

For specific causes of death, usual alcohol intake was associated with higher risks of IHD and stroke types (Figure 2), cancers of the oesophagus, liver, and stomach, ALD and liver cirrhosis, and self-harm **(appendix p18**). Associations were stronger for cancers pre-defined by IARC as alcohol-related (1.33, 1.27−1.41), compared with other cancers (1.09, 1.04−1.13), and for causes pre-defined by WHO as alcohol-related (1.25, 1.21−1.28), compared with other causes (1.09, 1.05−1.12) (Figure 2; **appendix p21**). The patterns of association were unaltered in sensitivity analyses to further address reverse causation and residual confounding (**appendix p22**).

Among women, ex- and non-drinkers had higher risks of deaths from most causes compared with occasional/moderate drinkers, but among the few current drinkers, usual alcohol intake was only significantly associated with CVD mortality (1.50, 1.06−2.13)) (**appendix p23**).

### Associations of genotype-predicted alcohol intake with mortality in men

Among genotyped participants, there were 23,457 deaths (13,177 in men, 10,280 in women) (**appendix p17**). In contrast with the J- or U-shaped associations seen with self-reported alcohol consumption, among men mortality risks increased linearly across the range of genotype-predicted mean alcohol intake for CVD (HR per 100 g/week 1.15, 95% CI 1.10−1.19), liver diseases (1.31, 1.02−1.69), and all-causes (1.07, 1.05−1.10), in pooled within-area analyses adjusted for age-at-risk and genomic PCs (Figure 1; Table 2). The genetic results were somewhat weaker than the corresponding estimates in the observational analyses (e.g. 1.07 vs. 1.18 per 100 g/week for all-cause mortality). There were no associations with respiratory, other medical or non-medical causes of death. Although there was no association of genotype-predicted alcohol intake with overall cancer mortality (1.01, 0.97−1.06), there was a positive association with the aggregated alcohol-related cancers (1.12, 1.04−1.21) (Figure 2), including cancer of the oesophagus (1.16; 1.02−1.31) (Table 2). In contrast to the positive association in conventional analyses, there was no associations with the aggregated other cancers (0.91; 0.91−1.01).

Among types of CVD death, genotype-predicted alcohol intake was associated with higher risks of IS (1.12, 1.00−1.25), ICH (1.20, 1.13−1.28), and total stroke (1.18, 1.12−1.24). There were no significant associations with MI (1.05, 0.96−1.15) or overall IHD (1.06, 0.99−1.14), although the trend was positive in both cases (Figure 2).

Genotype-predicted alcohol intake was associated with the aggregated WHO alcohol-related causes (1.13, 1.09−1.16), but not with other causes of death (1.00, 0.97−1.04) (**appendix p21**).

Sensitivity analyses, including those which excluded the highest category of genotype-predicted alcohol intake, did not materially alter the main genotypic findings, and although the magnitude of the excess risks varied e.g. 7-10% per 100 g/week for all-cause mortality, the 95% CIs all overlapped (**appendix p24-26**).

*ALDH2*-rs671 GG was associated with higher risks of CVD and all-cause mortality, and *ADH1B*-rs1229984 GG with higher risks of alcohol-related cancer, CVD and all-cause mortality, compared with GA genotypes (**appendix p27-28**).

There were interactions between *ALDH2*-rs671 genotype and self-reported alcohol intake for alcohol-related cancers, other cancers, and all-cause mortality, with higher risks among male drinkers with AG compared with GG genotypes (**appendix p29**). When cancers were excluded, the interaction for all-cause mortality was null. There were no interactions with *ADH1B*-rs1229984 (**appendix p30**). The HR per 100 g/week genotype-predicted alcohol intake for all-cause mortality excluding cancers was 1.10 (1.07−1.13) (**appendix p21**).

### Genetic associations in women to assess pleiotropy

Among women, using the same genotype-area categories as in men, there were no excess risks of cause-specific mortality (**appendix p31**). There were, however, lower risks of deaths from other medical causes (n=868 deaths; 0.85, 0.78-0.92), all-causes (n=10,057; 0.97, 0.94-0.99), and colorectal cancer, lung cancer, and diabetes. Genotype-predicted risks differed substantially between men and women, with excess risks among men for alcohol-related cancer, CVD (including stroke types), liver, and all-cause mortality (Figures 1 & 2). For both individual variants, there were excess risks among men for CVD and all-cause mortality, compared with women (**appendix p27-28**). Restricting the genetic analyses to 292,724 women non-smokers did not alter the main findings (**appendix p32**).

### Associations of polygenic score-predicted alcohol intake with mortality

Among men, mean alcohol intake varied from 57-162 g/week across quintiles of a polygenic score, with no associations with potential confounders apart from small differences in fruit intake and smoking (**appendix p33**). Polygenic score-predicted alcohol intake was associated with higher mortality risks from alcohol-related cancers (1.26, 1.06−1.49), CVD (1.18, 1.09−1.27), including stroke (1.22, 1.11−1.35) and IHD (1.16, 1.01−1.33), and all causes (1.10, 1.05−1.16) (**appendix p34-35**). Risks of liver disease deaths (n=68) were higher (1.68, 0.99−2.86) but not significantly. Among women, there were no associations, and the polygenic score only predicted small alcohol intake differences (3-6 g/week).

Among men, the associations with mortality from alcohol-related cancers, CVD, WHO alcohol-related causes, and all-causes were generally linear and uniform, with similar LACE estimates across five strata with mean alcohol intakes from 4-371 g/week (**appendix p37-38**). Although LACE estimates varied when alcohol was not log-transformed, or using the residual method to define strata, these factors may cause bias, particularly for alcohol intake which has an irregular distribution.^24^

## Discussion

In this large prospective study of Chinese adults, using a strong genetic instrument to predict alcohol intake, we demonstrated that genotype-predicted alcohol intake was associated with higher risks of mortality from CVD, particularly stroke, certain cancers, liver diseases, and all-causes. In contrast to the J-shaped associations seen in conventional observational analyses, there was no genetic evidence for a protective effect of moderate drinking for major causes of death, including stroke and IHD, or overall mortality. For stroke, mortality risks increased linearly with amount of genotype-predicted alcohol intake, while for IHD mortality, there was a non-significant positive trend. Moreover, analyses among Chinese women, who had very low intakes of alcohol, showed that the excess mortality hazards among men were likely to be chiefly due to alcohol itself, rather than to genetic pleiotropy.

Over the past several decades, numerous prospective studies have reported the lowest mortality risks among moderate drinkers (i.e., 1-2 drinks per day), driven mainly by CVD deaths, in particular IHD.^5,6,9,25^ In a combined analysis of 83 prospective studies, involving mainly Western populations and ~48,000 deaths, the adjusted all-cause mortality risks were higher in ex- and non-drinkers and heavier drinkers, compared with moderate drinkers, and among current drinkers, risks did not increase until a threshold of ~2 drinks/day.^5^ While stroke mortality increased with higher alcohol intake, associations with IHD were less clear, with potentially different patterns for fatal and non-fatal events.^5^ In the present study, with ~20,000 deaths in Chinese men,, we found similar lower risks among moderate drinkers, for mortality overall, and for most major causes, including IHD and stroke, despite rigorous approaches to control for reverse causation and residual confounding. Among male drinkers, however, there were continuous positive associations with major causes of death, apart from respiratory diseases, even at lower intake levels, with no evidence of a threshold below which alcohol was unrelated to risk.

In recent years, MR has been used to evaluate the likely causal relevance of alcohol for different diseases, but although a few previous MR studies have reported higher risks of all-cause mortality with alcohol intake, they did not assess cause-specific mortality, or evaluate causal relevance at different levels of intake.^12,14,26^ A study including 13,700 deaths in UK Biobank, reported higher risks of all-cause mortality associated with each additional drink/day, using *ADH1B*-rs1229984 (OR 1.44, 95% CI 1.09-1.90) or a 25-SNP score (1.31, 1.08-1.59).^12^ A study in Australian men with 1,329 deaths reported 47% higher all-cause mortality risk for *ADH1B*-rs1229984 GG compared with GA/AA genotypes (who drank less).^26^ In the present genetic analyses, with 12,939 deaths in men, there was 17% (10-24%) higher risk of all-cause mortality for *ADH1B*-rs1229984 GG compared with GA genotypes.

In East Asians, where the common *ALDH2*-rs671 variant is a strong determinant of alcohol intake, previous studies, including CKB, have assessed the causal relevance of alcohol in incident risk of CVD, cancer, and other diseases.^4,13,27,28^ For cause-specific mortality, however, evidence from prospective studies is limited. A study with 2037 deaths in Chinese men reported a nominal trend for higher all-cause mortality with alcohol intake predicted by *ALDH2*-rs671.^14^ In Biobank Japan participants (31,403 deaths) both *ALDH2*-rs671 and *ADH1B*-rs1229984 A alleles were weakly associated with lower all-cause mortality, but the findings were adjusted for alcohol so causal relevance could not be properly evaluated.^29^ In the present MR study of ~23,000 deaths (~13,000 in men), with a genetic instrument that predicted a 60-fold difference in mean alcohol intake in men, we found a linear dose-response causal association of alcohol intake with risks of death from all-causes, CVD (particularly stroke), certain cancers (e.g., oesophageal) and liver diseases, consistent with well-established hazards considered by WHO to be alcohol-related.^2^ For IHD mortality, there was no genetic evidence of any apparent protective effects of moderate drinking, if anything there was a positive trend towards higher risks with alcohol intake, which differs somewhat from the null association with non-fatal IHD.^4,13^.

Given the very low alcohol consumption among women in the study, there was a unique opportunity to assess pleiotropy of the genetic variants, which provided strong support that the excess risks for CVD, certain cancers, liver and overall deaths in men were due to alcohol itself. Although the *ALDH2*/*ADH1B* instrument had inverse associations in women for some outcomes, these were modest, and if anything would have attenuated the genetic associations in men towards the null. For causes pre-defined as unrelated to alcohol, the null genetic associations in men, in contrast to positive associations with self-reported alcohol intake, indicate that the genetic approach is robust to confounding. Moreover, the lower genetic risk estimates, compared with the conventional dose-response estimates, also suggest potential uncontrolled residual confounding in the conventional analyses.

Estimation of the alcohol-attributable disease burden generally uses evidence from observational studies which may not always reflect causal associations (e.g. the apparently lower risks of CVD with moderate drinking), and large-scale randomised trial evidence is unavailable.^1,3^ We demonstrate that alcohol itself is likely to be causally associated with deaths from several major causes in a linear and graded manner, with no apparent protective effects of moderate drinking for major causes of death, including CVD. Based on the ~7% excess risks for overall mortality per 100 g/week genotype-predicted alcohol intake, and the reported mean alcohol intake levels among men in the study, we estimate that alcohol drinking accounted for ~7-8% of male deaths in this Chinese population. This is somewhat lower than that reported by other studies in China (e.g. ~12% of male deaths at age 40-70 years in the 2016 GBD report).^1,16^ In addition to differences in relative risk estimates, sex-, region-and age-specific drinking levels, and the proportions of deaths from different causes in different settings, could greatly affect the estimation of alcohol-attributed mortality in China (and elsewhere).^3^

Our study has several strengths, including a large number of deaths, use of strong genetic instruments, and ability to assess genetic pleiotropy. However, it also has limitations. First, we lacked statistical power to study the effects of alcohol on less frequent causes of death, (e.g. tuberculosis), causes only affecting women (e.g. breast cancer), or causes such as injuries which may relate to alcohol differently among younger people or in different social contexts.^1,3^ Second, our cohort study may have recruited disproportionately fewer heavy drinkers, or more healthy people who had survived to middle-age, leading to potential selection biases. Third, we did not assess associations of longitudinal drinking measurements with cause-specific mortality. Fourth, using the genetic methods available, we could not assess the causal relevance of drinking patterns (e.g. heavy drinking episodes or consumption with meals), and beverage types (e.g. wine compared with spirits) for cause-specific mortality. Finally, the causal estimates varied somewhat by the methodology used, and were lower than the estimates in conventional analyses. However, this variation was small, and different methods, including use of an alternative polygenic score, gave generally consistent findings.

This study has shown that alcohol use uniformly increases the risks of death overall, and from major causes including CVD, certain cancers and liver diseases, among Chinese men, with no evidence of protection conferred by moderate alcohol intake. Genetic evidence about the causal relevance of alcohol consumption for mortality from different causes, in populations of diverse ancestry and demography, can improve the estimation of the global harms of alcohol use. Evidence on the harms of alcohol use is important to inform and support public health strategies to reduce population levels of alcohol consumption. This has started to be reflected in policy changes in some countries, for example, Canada has recently introduced guidance for low-risk drinking at a threshold of 1-2 drinks/week,^30^ and new evidence from the present study may help accelerate the policy changes in other countries.

## Figures and Tables

**Table 1:** Baseline characteristics by alcohol drinking status, in men and women

					Current drinkers				
	Overall	Non-drinkers	Ex-drinkers	Occasional drinkers	All current drinkers	< 140 (men) / < 70 (women) g per week	140-279 (men) / ≥ 70 (women) g per week	280-419 (men) g per week	≥ 420 (men) g per week
**Men, N (%)**	210205	42779 (20.4)	18295 (8.7)	79231 (37.7)	69900 (33.3)	25093 (11.9)	18907 (9.0)	12832 (6.1)	13068 (6.2)
**Sociodemographic characteristics**									
Mean age, years (SD)	52.8 (10.9)	57.0 (11.1)	56.8 (10.3)	51.0 (10.8)	51.5 (10.2)	51.3 (10.9)	51.9 (10.2)	51 (9.6)	50.7 (9.5)
Urban, N (%)	91358 (43.5)	13974 (31.2)	7714 (41.1)	34645 (44.1)	35025 (50.0)	14730 (58.6)	9967 (52.7)	6257 (48.5)	4071 (31.2)
Education > 6 years, N (%)	121429 (57.8)	18770 (54.5)	8720 (56.7)	51809 (60.5)	42130 (57.6)	17618 (63.9)	11559 (60.1)	7259 (59.6)	5694 (55.7)
Household income >20,000 y/yr, N (%)	95937 (45.6)	17816 (42.0)	7935 (44.9)	34181 (46.7)	36005 (46.8)	13489 (53.1)	9769 (51.3)	6737 (49.6)	6010 (50.4)
**Lifestyle risk factors**									
Current smokers, %	128371 (61.1)	23063 (52.3)	10531 (60.4)	44943 (56.9)	49834 (71.7)	15849 (64.6)	13632 (72.1)	9818 (76.1)	10535 (79.6)
Regular fresh fruit intake ^a^, N (%)	48414 (23.0)	8638 (24.9)	4368 (25.3)	19467 (25.2)	15941 (21.1)	7606 (28.0)	4241 (22.0)	2299 (18.9)	1795 (16.4)
Mean physical activity, MET-h/d (SD)	22.0 (15.3)	21.1 (15.1)	20.3 (14.5)	22.5 (15.6)	22.2 (15)	22.6 (14.5)	23.1 (14.9)	23.1 (15.4)	22.5 (15.2)
Mean SBP, mmHg (SD)	132.8 (20)	132 (21.5)	134.1 (21.5)	131 (18.8)	134.3 (19.8)	131.8 (18.9)	134.3 (19.8)	136 (20)	137.7 (20.7)
Mean BMI, kg/m^2^ (SD)	23.4 (3.2)	23.3 (3.2)	23.9 (3.4)	23.4 (3.2)	23.4 (3.2)	23.7 (3.2)	23.7 (3.2)	23.7 (3.2)	23.8 (3.2)
**Self-reported medical history, N (%)**									
Poor self-reported health	18741 (8.9)	4852 (12.8)	3453 (17.1)	6040 (7.7)	4396 (5.9)	1532 (6.5)	1185 (6.4)	764 (6.1)	915 (7.1)
Prior chronic disease ^b^	47547 (22.6)	11540 (27.4)	7441 (37.9)	15801 (21.2)	12765 (18.0)	5234 (19.9)	3421 (18.0)	2108 (17.3)	2002 (17.5)
									
**Women, N (%)**	302519	192333 (63.6)	2657 (0.9)	101285 (33.5)	6244 (2.1)	3224 (1.1)	3020 (1.0)	-	-
**Sociodemographic characteristics**									
Mean age, years (SD)	51.5 (10.5)	52.7 (10.7)	55.2 (9.4)	49.3 (9.9)	52.9 (10.3)	53 (10.7)	52.8 (9.9)	-	-
Urban, N (%)	134833 (44.6)	83001 (42.7)	708 (30.2)	48305 (48.1)	2819 (46.5)	1977 (60.9)	842 (29.0)	-	-
Education > 6 years, N (%)	130930 (43.3)	67035 (41.2)	799 (46.5)	60127 (49.0)	2969 (48.2)	1927 (49.6)	1042 (44.6)	-	-
Household income > 20,000 y/yr, N (%)	123095 (40.7)	82323 (38.0)	776 (44.8)	37538 (44.2)	2458 (47.0)	1593 (41.1)	865 (36.8)	-	-
**Lifestyle risk factors**									
Current smokers, N (%)	7151 (2.4)	3131 (1.9)	300 (5.4)	2740 (2.8)	980 (7.9)	243 (10.0)	737 (20.5)	-	-
Regular fresh fruit intake ^a^, N (%)	96133 (31.8)	52003 (30.0)	851 (42.7)	40726 (36.9)	2553 (39.1)	1681 (44.1)	872 (35.2)	-	-
Mean physical activity, MET-h/d (SD)	20.4 (12.8)	20.1 (13.3)	20.2 (11.1)	20.6 (11.7)	20.5 (11.6)	20 (11.5)	19.6 (11.7)	-	-
Mean SBP, mmHg (SD)	129.9 (22)	130.8 (22.5)	129.1 (23.2)	127.9 (20.5)	127.8 (21.6)	127.5 (20.9)	129.3 (22.1)	-	-
Mean BMI, kg/m^2^ (SD)	23.8 (3.5)	23.9 (3.5)	24 (3.5)	23.8 (3.4)	23.7 (3.4)	23.8 (3.4)	23.8 (3.4)	-	-
**Self-reported medical history, N (%)**									
Poor self-reported health	34350 (11.4)	22080 (12.6)	648 (21.5)	10987 (9.6)	635 (8.0)	299 (10.8)	336 (9.8)	-	-
Prior chronic disease ^b^	67276 (22.2)	44474 (23.3)	989 (33.1)	20417 (20.9)	1396 (19.9)	771 (23.9)	625 (21.9)	-	-

MET-h/d, metabolic equivalent of task per hour per day; SD, standard deviation; y/yr, yuan/year

Means and percentages are adjusted for the age and study area structure of the CKB population for the four drinking groups, and for the CKB drinker population for the weekly intake groups, using direct standardisation separately by sex.

a 4+ days/week;

b Chronic diseases included self-reported history of coronary heart disease, stroke, transient ischaemic attack, diabetes, tuberculosis, cirrhosis, hepatitis, rheumatoid arthritis, peptic ulcer, emphysema/chronic bronchitis, gallstone/gallbladder disease, rheumatic heart disease, and kidney disease.

**Table 2:** Genetic associations of alcohol intake with cause-specific mortality, in men

Cause of death	N	C1 HR (95% CI)	C2 HR (95% CI)	C3 HR (95% CI)	C4 HR (95% CI)	C5 HR (95% CI)	C6 HR (95% CI)	Per 100 g/week HR (95% CI)	p- value
**Infectious diseases**	163	1.00 (0.75-1.32)			0.83 (0.5-1.38)	1.38 (0.86-2.23)	1.47 (0.88-2.46)	1.22 (0.96-1.56)	0.11
Viral hepatitis	75	1.00 (0.69-1.45)	--	--	0.30 (0.12-0.76)	0.95 (0.43-2.09)	1.26 (0.54-2.91)	1.02 (0.64-1.62)	0.94
**Cancers**	3428	1.00 (0.87-1.15)	1.07 (0.94-1.23)	1.13 (1.04-1.24)	1.02 (0.92-1.12)	1.09 (0.97-1.23)	1.09 (1.00-1.20)	1.01 (0.97-1.06)	0.53
Oesophageal cancer	406	1.00 (0.55-1.81)	1.71 (1.27-2.31)	1.45 (0.97-2.16)	1.69 (1.37-2.08)	2.00 (1.35-2.95)	2.35 (1.70-3.23)	1.16 (1.02-1.31)	0.028
Colorectal cancer	225	1.00 (0.50-2.02)	1.71 (0.96-3.03)	1.74 (1.23-2.47)	2.05 (1.39-3.01)	1.62 (1.04-2.52)	2.40 (1.70-3.37)	1.05 (0.88-1.26)	0.59
Liver cancer	557	1.00 (0.71-1.40)	0.92 (0.66-1.27)	1.19 (0.96-1.47)	1.02 (0.80-1.32)	1.22 (0.92-1.60)	1.29 (1.02-1.64)	1.07 (0.95-1.19)	0.28
Stomach cancer	498	1.00 (0.72-1.38)	1.16 (0.88-1.53)	1.05 (0.84-1.32)	0.87 (0.68-1.12)	0.73 (0.48-1.10)	0.96 (0.75-1.23)	0.94 (0.84-1.07)	0.36
Lung cancer	1003	1.00 (0.79-1.27)	0.99 (0.74-1.33)	0.97 (0.84-1.11)	0.83 (0.68-1.03)	0.93 (0.77-1.13)	0.82 (0.70-0.96)	0.93 (0.86-1.01)	0.091
Alcohol-related cancers	1274	1.00 (0.77-1.30)	1.24 (1.01-1.52)	1.40 (1.19-1.65)	1.29 (1.11-1.50)	1.41 (1.17-1.72)	1.69 (1.44-1.99)	1.12 (1.04-1.21)	0.0029
Other (non-alcohol) cancers	2154	1.00 (0.85-1.18)	1.03 (0.87-1.23)	1.02 (0.93-1.13)	0.92 (0.81-1.05)	0.97 (0.84-1.12)	0.89 (0.80-0.99)	0.96 (0.91-1.01)	0.10
**Cardiovascular diseases**	6109	1.00 (0.91-1.10)	1.06 (0.97-1.15)	1.05 (0.98-1.11)	1.05 (0.96-1.13)	1.19 (1.10-1.28)	1.52 (1.39-1.67)	1.15 (1.10-1.19)	<0.0001
Hypertensive heart disease	152	1.00 (0.80-1.24)	--	--	0.69 (0.38-1.24)	1.18 (0.67-2.06)	1.44 (0.96-2.16)	1.21 (0.98-1.51)	0.082
Ischaemic heart disease	2363	1.00 (0.85-1.17)	1.10 (0.95-1.26)	1.13 (1.02-1.25)	1.03 (0.91-1.18)	1.11 (1.00-1.24)	1.35 (1.15-1.57)	1.06 (0.99-1.14)	0.079
Myocardial infarction	1382	1.00 (0.82-1.21)	1.09 (0.93-1.28)	1.09 (0.96-1.25)	1.04 (0.88-1.23)	1.14 (0.97-1.33)	1.40 (1.12-1.76)	1.05 (0.96-1.15)	0.25
Stroke	3285	1.00 (0.88-1.14)	1.00 (0.89-1.12)	1.00 (0.92-1.09)	1.05 (0.94-1.17)	1.26 (1.13-1.4)	1.61 (1.43-1.82)	1.18 (1.12-1.24)	<0.0001
Ischaemic stroke	929	1.00 (0.76-1.32)	0.88 (0.71-1.10)	1.12 (0.92-1.35)	1.02 (0.85-1.23)	1.22 (1.00-1.48)	1.52 (1.19-1.94)	1.12 (1.00-1.25)	0.047
Intracerebral haemorrhage	2185	1.00 (0.86-1.16)	1.03 (0.90-1.19)	0.97 (0.88-1.07)	1.02 (0.88-1.18)	1.26 (1.10-1.43)	1.71 (1.46-1.99)	1.20 (1.13-1.28)	<0.0001
**Respiratory diseases**	1513	1.00 (0.84-1.19)	1.03 (0.85-1.24)	0.89 (0.79-1.00)	0.93 (0.79-1.09)	1.01 (0.85-1.20)	0.93 (0.81-1.08)	0.99 (0.93-1.05)	0.81
Pneumonia	176	1.00 (0.78-1.28)	--	--	1.11 (0.78-1.59)	0.97 (0.63-1.48)	1.77 (1.01-3.12)	1.11 (0.82-1.51)	0.49
COPD	1214	1.00 (0.83-1.21)	1.07 (0.85-1.33)	0.91 (0.80-1.03)	0.89 (0.74-1.08)	1.05 (0.86-1.27)	0.90 (0.76-1.05)	0.99 (0.93-1.06)	0.82
**Liver diseases**	110	1.00 (0.65-1.54)	--	--	1.33 (0.80-2.20)	1.20 (0.65-2.23)	2.58 (1.52-4.38)	1.31 (1.02-1.69)	0.035
ALD and liver cirrhosis	92	1.00 (0.60-1.66)	--	--	1.45 (0.85-2.50)	1.10 (0.54-2.24)	3.12 (1.79-5.46)	1.34 (1.02-1.76)	0.034
**Other medical causes**	881	1.00 (0.76-1.31)	1.33 (1.07-1.65)	1.09 (0.90-1.32)	1.19 (0.99-1.43)	1.45 (1.16-1.81)	1.34 (1.08-1.66)	1.05 (0.96-1.15)	0.30
Diabetes mellitus	231	1.00 (0.57-1.75)	1.68 (1.08-2.63)	1.13 (0.79-1.60)	1.67 (1.15-2.41)	1.35 (0.86-2.13)	1.82 (1.24-2.67)	1.00 (0.84-1.18)	0.96
Renal diseases	119	1.00 (0.79-1.27)	--	--	0.67 (0.38-1.20)	1.59 (1.01-2.51)	0.90 (0.45-1.81)	1.08 (0.81-1.43)	0.61
Ill-defined and unknown causes	174	1.00 (0.79-1.26)	--	--	1.03 (0.77-1.38)	1.69 (1.04-2.74)	0.82 (0.40-1.71)	1.00 (0.72-1.41)	0.99
**Non-medical causes**	717	1.00 (0.76-1.32)	1.09 (0.83-1.44)	0.92 (0.77-1.11)	0.99 (0.79-1.23)	1.19 (0.93-1.53)	1.00 (0.81-1.22)	1.02 (0.93-1.12)	0.64
Transport accidents	281	1.00 (0.63-1.58)	0.92 (0.55-1.53)	1.04 (0.79-1.38)	0.99 (0.69-1.41)	0.91 (0.60-1.38)	0.91 (0.67-1.23)	0.96 (0.83-1.10)	0.52
Falls	154	1.00 (0.76-1.32)	--	--	1.00 (0.63-1.57)	1.62 (0.90-2.90)	0.99 (0.63-1.55)	1.01 (0.81-1.24)	0.95
Self-harm	86	1.00 (0.68-1.47)	--	--	1.13 (0.53-2.44)	1.35 (0.74-2.43)	2.00 (1.05-3.81)	1.13 (0.85-1.50)	0.40
**All causes**	12939	1.00 (0.94-1.07)	1.06 (1.00-1.13)	1.04 (1.00-1.09)	1.02 (0.97-1.08)	1.15 (1.08-1.21)	1.23 (1.16-1.30)	1.07 (1.05-1.10)	<0.0001
WHO alcohol-related causes	8218	1.00 (0.92-1.09)	1.06 (0.98-1.14)	1.05 (0.99-1.11)	1.06 (0.99-1.14)	1.16 (1.09-1.24)	1.44 (1.34-1.55)	1.13 (1.09-1.16)	<0.0001
Other causes	4721	1.00 (0.90-1.11)	1.10 (0.98-1.23)	1.04 (0.97-1.11)	0.97 (0.89-1.06)	1.16 (1.05-1.28)	1.01 (0.93-1.10)	1.00 (0.97-1.04)	0.91

Cox models were stratified by age-at-risk and study areas, and were adjusted for genomic national principal components. The slope per 100 g/week and p-value was obtained within areas, adjusted for age-at-risk and genomic regional principal components, and meta-analysed with IVWMA. The partial F statistic for genotype-predicted alcohol intake categories within each area ranged from 34-783 (1752 overall), and the partial r2 ranged from 0.012-0.225 (0.136 overall). For cause-specific mortality with fewer than 200 deaths, C1-C3 were combined as one genetic category.

## Data Availability

The CKB is a global resource for the investigation of lifestyle, environmental, blood biochemical and genetic factors as determinants of common diseases. The CKB Collaboration Group is committed to making the cohort data available to the scientific community worldwide to advance knowledge about the causes, prevention, and treatment of disease. For detailed information on what data are currently available to open access users and how to apply for data, visit https://www.ckbiobank.org/data-access. Researchers who are interested in obtaining the raw data from the CKB study that underlines this paper should contact ckbaccess@ndph.ox.ac.uk. A research proposal will be requested to ensure that any analysis is performed by bona fide researchers and, where data are not currently available to open access researchers, is restricted to the topic covered in this Article.

## Research in context

### Evidence before this study

Moderate alcohol intake has been associated with lower risks of mortality overall and from certain specific diseases, in particular, IHD. However, these associations may be largely non-causal as conventional observational studies of alcohol use are susceptible to bias from reverse causation and residual confounding. Genetic evidence from Mendelian randomisation studies, in particular using the *ALDH2*-rs671 and *ADH1B*-rs1229984 variants which strongly affect alcohol intake and are common in East Asian populations, can help assess the causal relevance of alcohol intake for cause-specific mortality.

The genetic evidence on alcohol consumption and mortality, was ascertained by searching PubMed from database inception to 25 February 2023 using the following search terms (title/abstract): ((Alcohol AND Mendelian) or (ALDH2 or ADH1B or rs671 or rs1229984 or aldehyde dehydrogenase or alcohol dehydrogenase)) AND (mortality or death or fatal), and reviewing bibliographies within the identified publications.

Two previous MR studies of alcohol and mortality in European ancestry populations, and one in Chinese men, reported that higher alcohol intake was associated with higher risks of all-cause mortality. However, these studies did not assess causal relevance across a wide range of alcohol intakes, and did not evaluate effects on cause-specific mortality.

### Added value of this study

The present prospective study used both conventional and genetic approaches within the same population. The genetic analyses minimised artefacts of confounding and reverse causation, and assessed potential causal relevance across a wide range of alcohol levels, from negligible, to moderate and heavy intakes. Among Chinese men, conventional observational analyses demonstrated characteristic J-shaped associations of self-reported alcohol intake categories with overall and cause-specific mortality, with highest risks among ex-, non-, and heavy drinkers, and lowest risks among moderate drinkers, consistent with findings from similar studies in Western populations.

Genetic analyses, using two genetic variants that predicted a 60-fold difference in mean intake from 4 g/week in the lowest to 255 g/week in the highest category, showed that higher alcohol intake was associated with a linear dose-response increase in risks of death overall and from certain cancers, CVD and liver diseases. There were no genetic associations with respiratory or non-medical (mainly accidents and injuries) causes of death. There was no genetic evidence that moderate alcohol intake (i.e. 10-20 g/day) had substantial protective effects for cause-specific or overall mortality, including for IHD deaths. In separate genetic analyses using a polygenic score to predict alcohol intake, there were similar and apparently linear associations with mortality overall, and from alcohol-related cancers and CVD, across different alcohol intake levels.

Alcohol intake was extremely low among women in the study, and the genetic variants had little effect on mortality overall or from specific causes, suggesting that the higher risks in men were chiefly mediated by alcohol, rather than by any pleiotropic effects of the genotypes studied.

### Implications of all the available evidence

Genetic studies, in East Asian ancestry populations in particular, have helped to reliably clarify the causal relevance of alcohol intake with mortality. Although this question has been extensively studied using conventional observational approaches, these methods have been unable to fully account for biases. The genetic evidence provides strong support for causal harmful effects of alcohol use with risks of deaths from CVD, cancer, liver disease and all-causes. There is no genetic evidence of any beneficial effects of moderate drinking compared with not drinking, for any causes of death, including CVD. These genetic studies which assess causal relevance have improved our understanding of the adverse effects of alcohol use on mortality, particularly at lower intake levels. This can improve estimation of the regional and global burden of alcohol use, and inform public health policies to address the risks of moderate as well as heavier drinking.
